# Supplementary material for: Neuronal DSCAM regulates the peri-synaptic localization of GLAST in Bergmann glia for functional synapse formation
Source: Nat Commun. 2024 Feb 1;15:458. doi: 10.1038/s41467-023-44579-z (PMC10834496; doi:10.1038/s41467-023-44579-z)
Supplement: Supplementary file 1 — Supplementary Information [file 41467_2023_44579_MOESM1_ESM.pdf]

Supplementary Information for

Neuronal DSCAM regulates the peri-synaptic localization of GLAST in Bergmann glia for functional synapse formation

Ken-ichi Dewa, Nariko Arimura, Wataru Kakegawa, Masayuki Itoh, Toma Adachi, Satoshi Miyashita, Yukiko U. Inoue, Kento Hizawa, Kei Hori, Natsumi Honjoya, Haruya Yagishita, Shinichiro Taya, Taisuke Miyazaki, Chika Usui, Shoji Tatsumoto, Akiko Tsuzuki, Hirotomo Uetake, Kazuhisa Sakai, Kazuhiro Yamakawa, Takuya Sasaki, Jun Nagai, Yoshiya Kawaguchi, Masaki Sone, Takayoshi Inoue, Yasuhiro Go, Noritaka Ichinohe, Kozo Kaibuchi, Masahiko Watanabe, Schuichi Koizumi, Michisuke Yuzaki, and Mikio Hoshino*

*Correspondence to: nariko.arimura.a2@tohoku.ac.jp, hoshino@ncnp.go.jp

Contents

Supplementary Figure 1. Expression of *Dscam* transcripts in the P8 cerebellum and non-specific staining of DSCAM in *En1*^{Cre}*-cKO* cerebellum slices.

Supplementary Figure 2. Generation of ALFA tag knock-in mice via the CRISPR/Cas system.

Supplementary Figure 3. Overall brain structure of P30 *Dscam*$^{del17/del17}$ mice during development.

Supplementary Figure 4. Confirmation of cell and synapse constitution in the molecular layer of the P30 *Dscam*$^{del17/del17}$ cerebellum.

Supplementary Figure 5. Confirmation of cell constitution in the molecular layer of the P7, P15, and P30 *Dscam*$^{del17/del17}$ cerebellum.

Supplementary Figure 6. Data sets of CF-EPSCs and PF-EPSCs.

Supplementary Figure 7. Expression of *Slc1a3* transcripts and GLAST protein in the *Dscam*$^{del17/del17}$ cerebellum.

Supplementary Figure 8. Analyses of the expression of CF translocation-related genes via whole-cerebellum RNA-seq analyses and Purkinje cell-selective prominent expression.

Supplementary Figure 9. No prominent change in the downstream molecules of mGluR1 in the *Dscam*$^{del17/del17}$ cerebellum.

Supplementary Figure 10. Morphological analysis of the PF synapses in the *Dscam*$^{del17/del17}$ cerebellum.

Supplementary Figure 11. Co-immunoprecipitation and western blot assays using the synaptosomal fraction.

Supplementary Figure 12. Overall brain structure of *Dscam* cKO mice.

Supplementary Figure 13. Schematic diagram of the phenotype of *Dscam*-deficient mice and proposed molecular mechanisms.

Supplementary Table 1. Statistical analyses of major components in the *Dscam*$^{del17/del17}$ cerebellum.

Supplementary Table 2. A table summarising whether the experiment was performed in a blind (B), automatic measurement (Auto), or non-blind manner (NB).

Supplementary References

Supplementary Figures

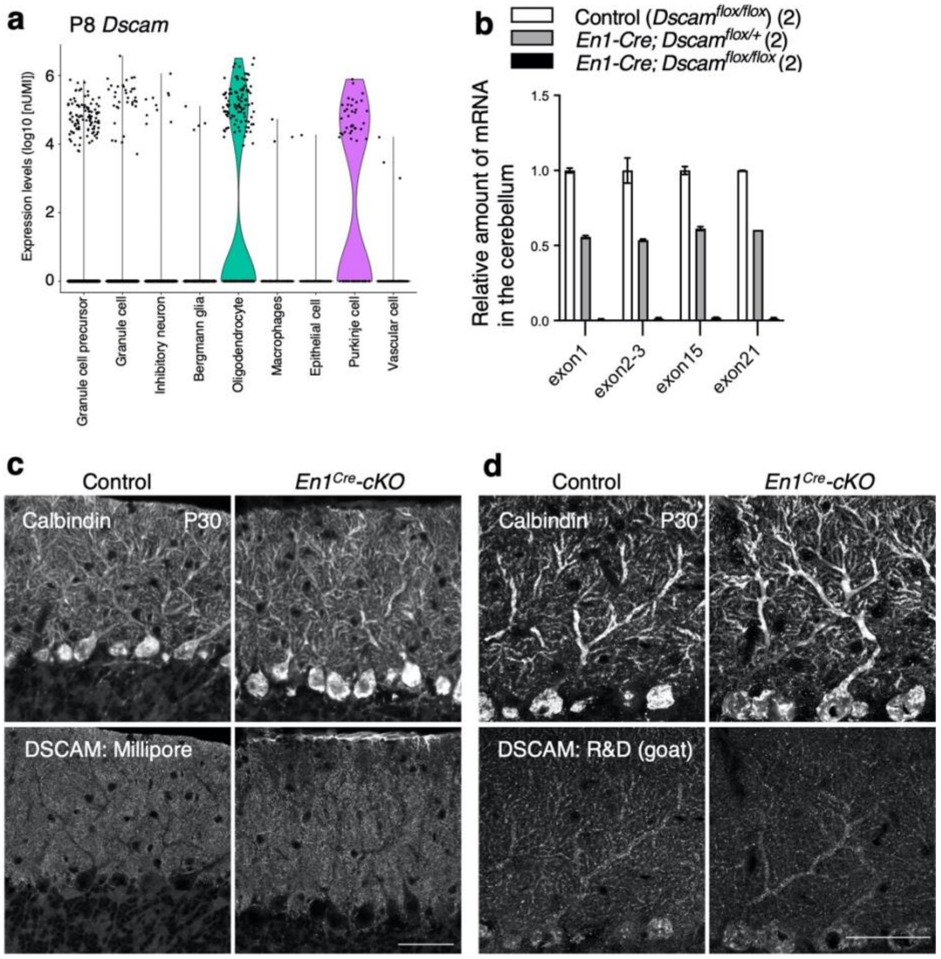

Supplementary Figure 1. Expression of *Dscam* transcripts in the P8 cerebellum and non-specific staining of DSCAM in *En1^{Cre}-cKO* cerebellum slices.

a, Violin plot of log10(nUMI) per profile across the nine cell types identified. Prominent expression was observed in oligodendrocytes and Purkinje cells in the P8 cerebellum[1]. UMI, unique molecular identifier.

b, Confirmation of the loss of *Dscam* transcripts in the cerebellum of *En1^{Cre}-cKO* mice. RT-PCR was performed using the primer set targeting exon1, exon2 to 3, exon15, or exon21 of *D*scam. Data represent mean. The numbers in parentheses are mice count.

c–d, Immunohistochemical analyses using sagittal sections of *En1^{Cre}-cKO* (right) and the control (*Dscam$^{flox/flox}$*) littermate (left) at P30. Two antibodies against DSCAM were used in this study. (**c**) Anti-DSCAM antibody produced in mice (DS2-176; Millipore). (**d**) Anti-DSCAM antibody produced in goat (AF3666; R&D Systems). Several immunohistochemical conditions, including different antibody dilutions (1:100–1:20,000), permeabilising agents (TritonX-100 and Saponin), blocking buffers (normal donkey serum, BSA, or skim milk used alone or in combination), fixation conditions (4% or 2% PFA), target retrieval method (boiling sections for 20 min in Target Retrieval Solution, pH 6 [DAKO] or in HistVT One, pH 7.0 [Nacalai Tesque]) using PFA-perfused or paraffin-embedding slices were tested. However, we were unable to obtain any distinct signals to distinguish between control and *En1^{Cre}-cKO* mice, suggesting

that other antigens similar to DSCAM may be expressed in the cerebellum. The staining conditions for the sections presented in **c–d** are as follows: 1:1,000 antibody dilution, 10% normal donkey serum as blocking buffer, and 4% PFA fixation without target retrieval. Data are confirmed by three independent experiments. Scale bars, 50 µm.

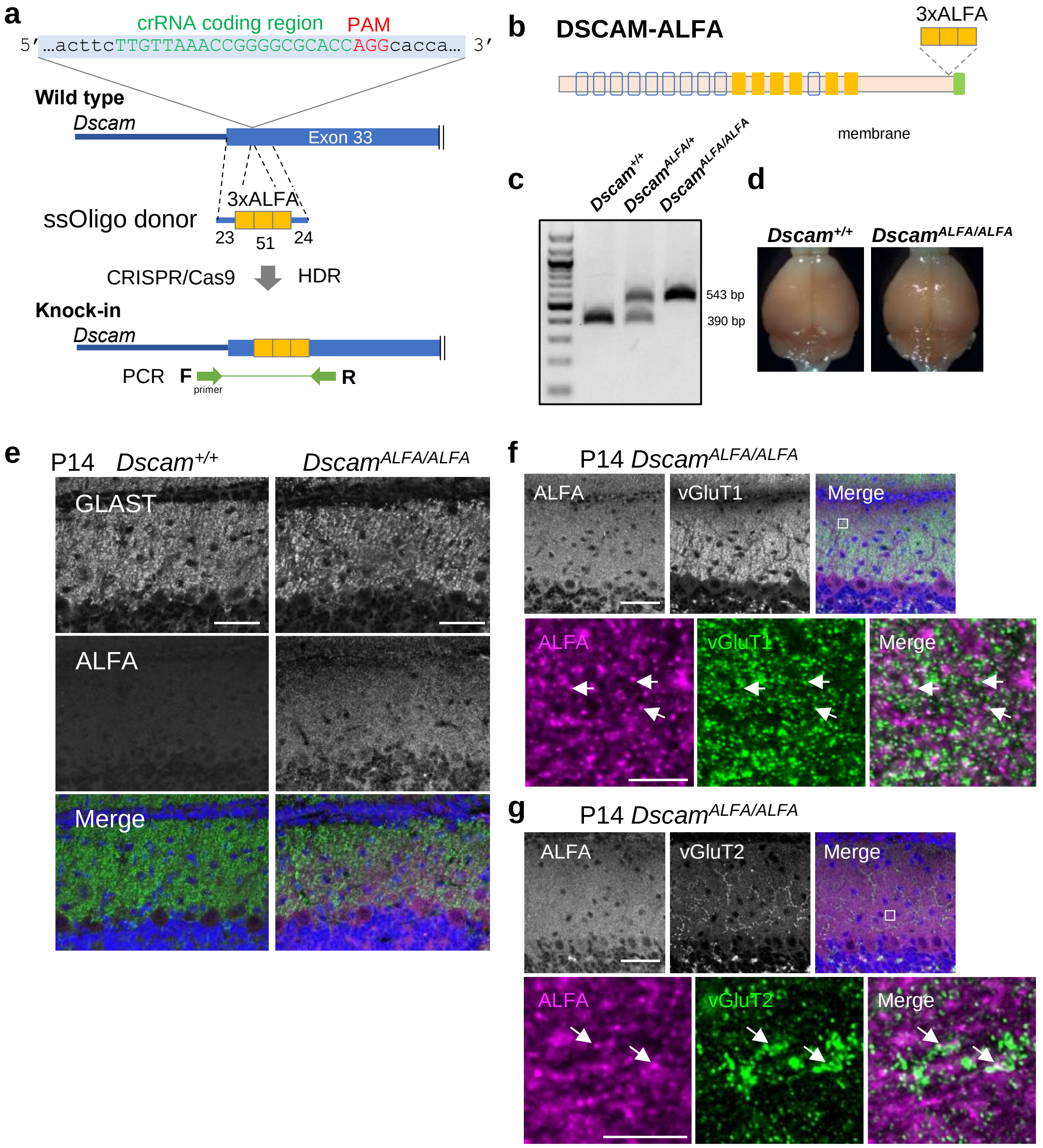

Supplementary Figure 2. Generation of ALFA tag knock-in mice via the CRISPR/Cas system.

a, Targeting strategy for the generation of *Dscam-ALFA tag* KI mice. The guide sequence used to target the cytoplasmic region of mouse *Dscam* was the same as that previously described for *Dscam*$^{PA/PA}$ mice[2]. The cleavage activity of crRNA has also been reported previously[2]. A single-stranded oligo donor encoding the 51 bp tandem ALFA tag sequence flanked by the 23-24 bp homology arms on both sides was prepared. Genotyping of the mouse tail genome was performed using forward (F) and reverse (R) primers. **b**, Diagram representing the domain structure of the DSCAM-ALFA protein. **c**, Gel image displaying PCR products amplified from the indicated genotypes. The PCR primers are shown in **a**. **d**, Overall brain structure of P30 mice for the indicated genotypes. Brains of mice that were heterozygous and homozygous for *Dscam*^{ALFA} were indistinguishable from those of control mice. (Photo credit: N. Arimura, Tohoku University) **e**, Confirmation of DSCAM-ALFA protein expression in the cerebellum via immunostaining using anti-ALFA nanobody and anti-GLAST antibodies. Data are confirmed by three

independent experiments. Scale bars, 50 µm. **f** and **g**, Immunostaining of endogenous ALFA-tagged DSCAM and presynaptic markers vGlut1 (**f**) or vGlut2 (**g**) in cerebellar slices from *Dscam*$^{ALFA/ALFA}$ mice on postnatal day 14. The higher magnification at the bottom shows the area surrounded by the white box. The white arrows indicate adjacent signals of DSCAM-ALFA and presynaptic markers. Data are confirmed by three independent experiments. Scale bars, upper 50 µm, lower 5 µm in **f** and **g**.

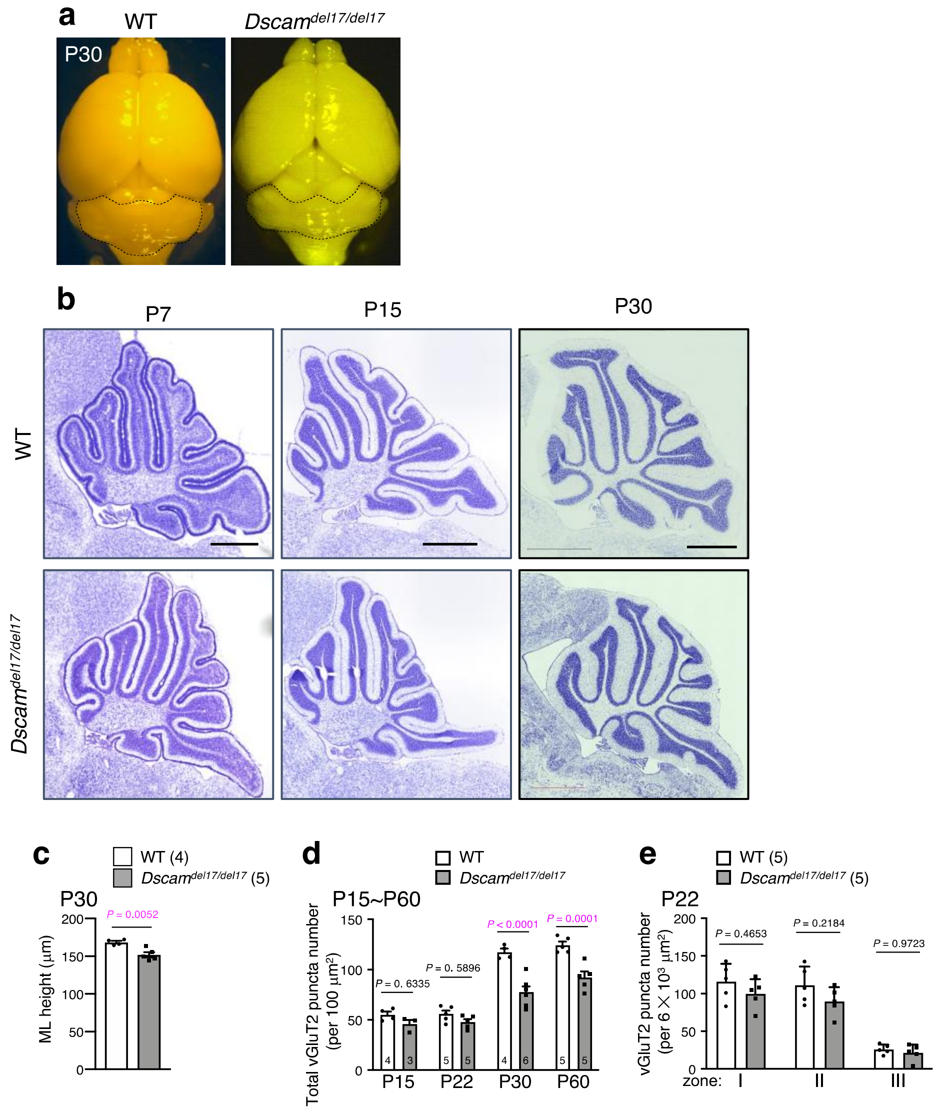

Supplementary Figure 3. Overall brain structure of P30 *Dscam*$^{del17/del17}$ mice during development.

a, P30 wild-type (WT) and *Dscam*$^{del17/del17}$ mouse brains. The cerebellum (dotted line) of *Dscam*$^{del17/del17}$ mice was pushed by the hypertrophic dorsal midbrain; however, its rough structure remained almost unchanged, as previously reported[3].

b, Nissl-stained sagittal sections of the P7, P15, and P30 control and *Dscam*$^{del17/del17}$ cerebellum did not reveal any gross anatomical differences. The distal portion of the caudal folia is sometimes abnormally long[3] from P7, probably because of hypertrophy of the dorsal midbrain[2]. Data are confirmed by three independent experiments. Scale bars, 1.0 mm.

c, In the P30 *Dscam*$^{del17/del17}$ cerebellum, thinning of the molecular layer in the sulcus of lobule 6 was observed. The numbers in parentheses are mice count. Data represent mean ± SEM; unpaired two-tailed *t*-test.

d, Developmental changes in the total number of vGluT2 puncta per 100 μm^2. The numbers in P15, P30, and P60 mice were the same as those demonstrated in Figure 2d. The numbers in each column indicate the number of mice examined. Data represent mean ± SEM; two-way ANOVA with multiple comparison.

e, Regional changes in the total number of vGluT2 puncta per 6×10^3 μm^2 in P22 mice. The zone is represented in Figure 2a. The numbers in parentheses are mice count. Data represent mean ± SEM; two-way ANOVA with multiple comparison.

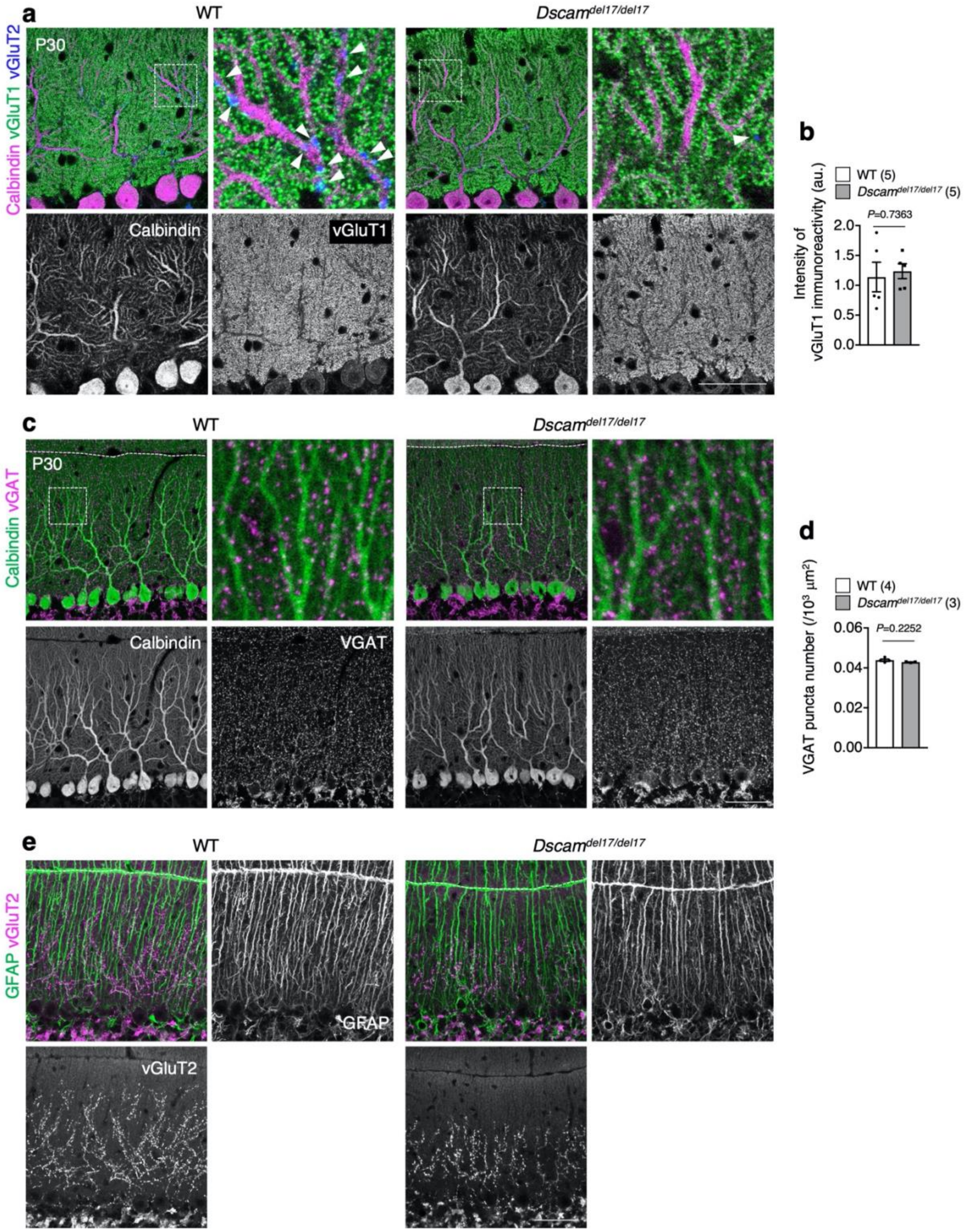

Supplementary Figure 4. Confirmation of cell and synapse constitution in the molecular layer of the P30 *Dscam$^{del17/del17}$* cerebellum.

a, Triple immunofluorescence for the Purkinje cell marker Calbindin (top, magenta), the parallel fibre (i.e., granule cell) synapse marker vGluT1 (top, green), and the climbing fibre synapse marker vGluT2 (top, blue) in the molecular layer of P30 *Dscam$^{del17/del17}$* and the control littermate (WT) cerebellum. The higher

magnification at the top right corner represents the area surrounded by the white dotted box. No obvious morphological anomalies were found in the major neuronal components of the *Dscam*$^{del17/del17}$ samples. Data are confirmed by three independent experiments. Scale bar, 50 µm.

b, Quantification of fluorescence intensity of VGluT1 in the WT or *Dscam*$^{del17/del17}$ cerebella at P30. The numbers in parentheses are mice count. Data represent mean ± SEM; unpaired two-tailed *t*-test.

c, Double immunofluorescence for the Purkinje cell marker Calbindin (top, green) and the inhibitory synapse marker VGAT (top, magenta). Higher magnification at the top right corner represents the area surrounded by the white dotted box. No obvious morphological anomalies were found in the major neuronal components of the *Dscam*$^{del17/del17}$ samples. Data are confirmed by three independent experiments. Scale bar, 50 µm.

d, Quantification of the number of VGAT-positive puncta (i.e., inhibitory synapses) in the WT or *Dscam*$^{del17/del17}$ cerebella at P30. The numbers in parentheses are mice count. Data represent mean ± SEM; unpaired two-tailed *t*-test.

e, Double immunofluorescence for the Bergmann glial marker GFAP (top, green) and the climbing fibre synapse marker vGluT2 (top, magenta). No obvious morphological anomalies were found in the major neuronal components of the *Dscam*$^{del17/del17}$ samples. Data are confirmed by three independent experiments. Scale bar, 50 µm.

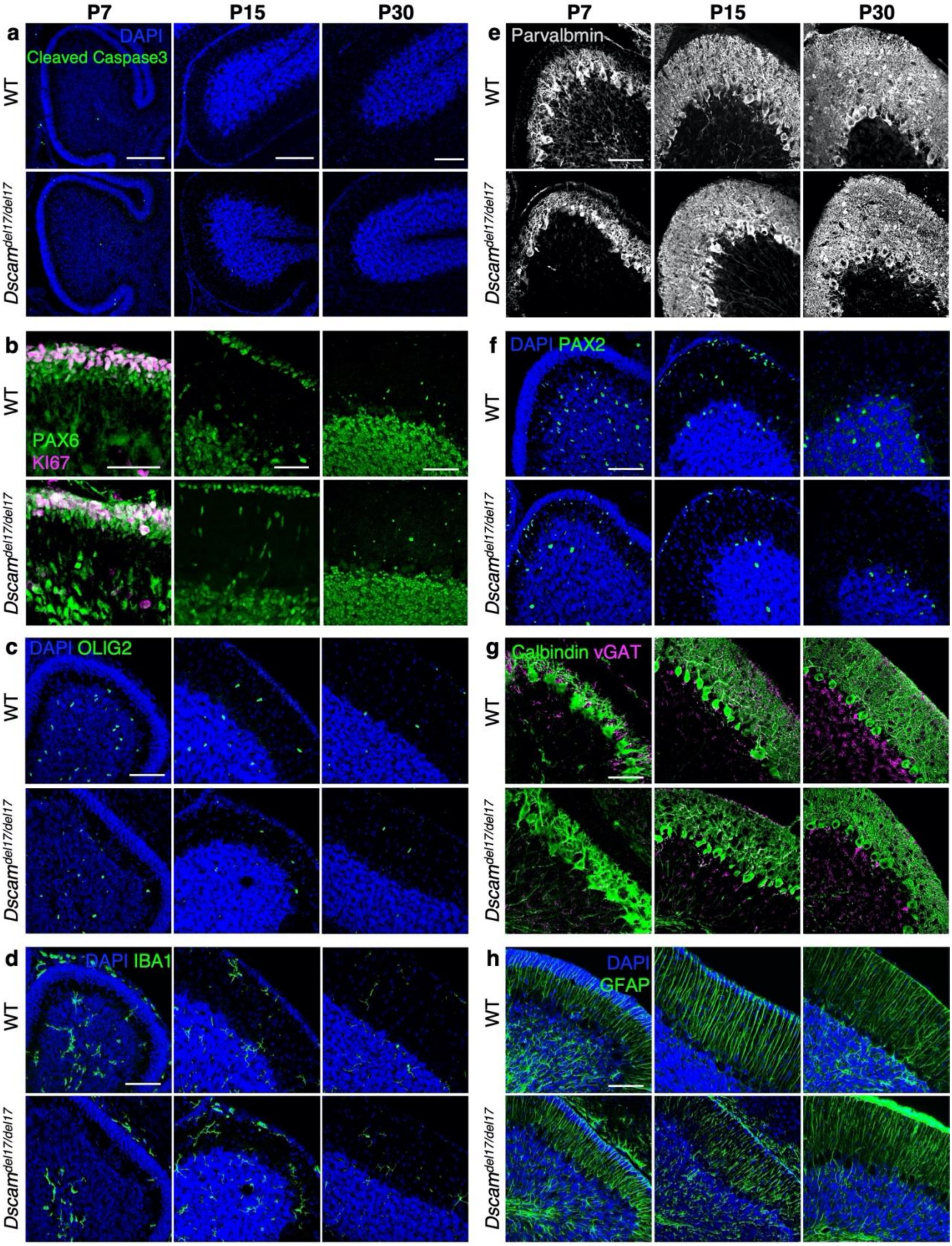

Supplementary Figure 5. Confirmation of cell constitution in the molecular layer of the P7, P15, and P30 *Dscam*$^{del17/del17}$ cerebellum.

a-h, Immunofluorescence for each type of cell in the developing cerebellum: the apoptotic cell marker Cleaved Caspase3 (**a**), the granule cell marker PAX6 (**b**), the proliferative cell marker KI67 **(b**), the oligodendrocyte and oligodendrocyte precursor cell marker OLIG2 (**c**), the microglia marker IBA1 (**d**), the Purkinje cell and interneuron marker Parvalbumin (**e**) the Golgi cell marker PAX2 (**f**), the Purkinje cell marker Calbindin (**g**), the inhibitory synapse marker vGAT (**g**), and the Bergmann glia marker GFAP (**h**) in the molecular layer of P7, P15, and P30 *Dscam*$^{del17/del17}$ and the control littermate (WT) cerebellum. The data of statistical analysis using these immunohistochemical images are summarised in Supplementary Table 1. The number of OLIG2-positive cells was significantly reduced in P7 but not in P15 and P30 *Dscam*$^{del17/del17}$ mice. Otherwise, no obvious morphological anomalies were found in the major neuronal components of the *Dscam*$^{del17/del17}$ samples. Data are confirmed by three independent experiments. Scale bars in **a**, 150 µm; in **b**, 40 µm in P7 and P15, 80 µm in P30; in **c**-**h**, 80 µm.

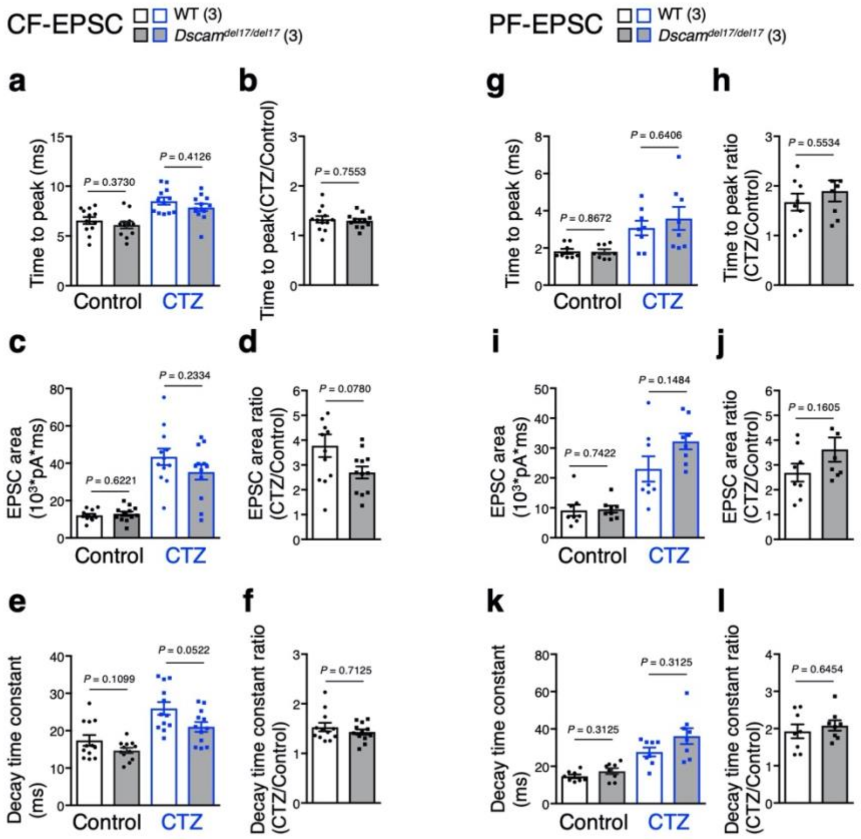

Supplementary Figure 6. Data sets of CF-EPSCs and PF-EPSCs.

a–**l**, Each parameter in CF-EPSCs (**a**–**f**) and PF-EPSCs (**g**–**l**) was examined: time to peak (**a** and **g**), time to peak ratio (**b** and **h**), EPSC area (**c** and **i**), EPSC area ratio (**d** and **j**), decay time constant (**e** and **k**), and decay time constant ratio (**f** and **l**) during P28 to P32. There was no significant quantitative difference in these parameters between the wild-type (WT) and *Dscam*$^{del17/del17}$ mice. The numbers in parentheses are mice count. Data represent mean ± SEM; two-tailed Mann-Whitney *U* test. CF, climbing fibre; PF, parallel fibre; EPSC, excitatory postsynaptic current; CTZ, cyclothiazide.

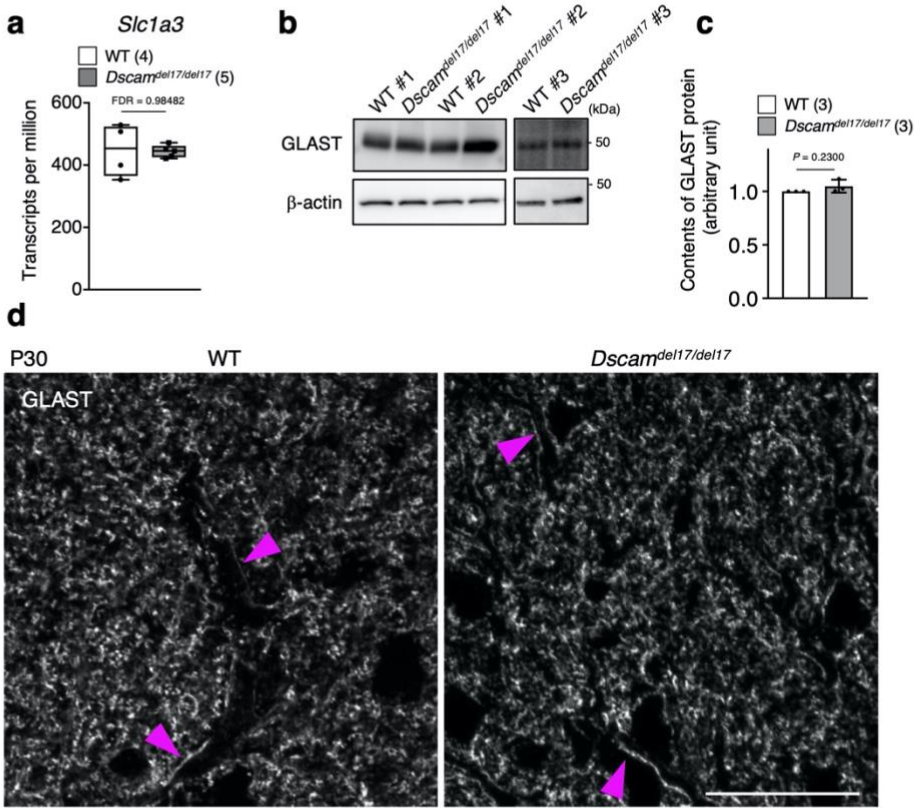

Supplementary Figure 7. Expression of *Slc1a3* transcripts and GLAST protein in the *Dscam*$^{del17/del17}$ cerebellum.

a, Whole-cerebellum RNA-seq analyses using *Dscam*$^{del17/del17}$ and control (WT) littermate mice revealed that the expression of *Slc1a3* (gene name of GLAST) mRNA was unchanged in *Dscam*$^{del17/del17}$ mice. The numbers in parentheses are mice count. Data represent mean ± SEM. Data were analysed via DEseq2. WT, wild-type; FDR, false-discovery rate.

b, Quantitative western blot analyses using GLAST antibody and P30 WT and *Dscam*$^{del17/del17}$ cerebella revealed that there was no significant quantitative difference between WT and *Dscam*$^{del17/del17}$ mice (*n* = 3).

c, Quantification of GLAST protein via western blot analyses shown in **b**. The numbers in parentheses are mice count. Data represent mean ± SEM; unpaired two-tailed *t*-test.

d, Immunohistochemical analyses using GLAST antibody and the sagittal section of P30 WT and *Dscam*$^{del17/del17}$ cerebella revealed that the densities and features were almost normal in *Dscam*$^{del17/del17}$ mice. The pink arrowheads indicate the elongated GLAST label along the primary dendrite shaft of a Purkinje cell. Data are confirmed by three independent experiments. Scale bar, 50 μm.

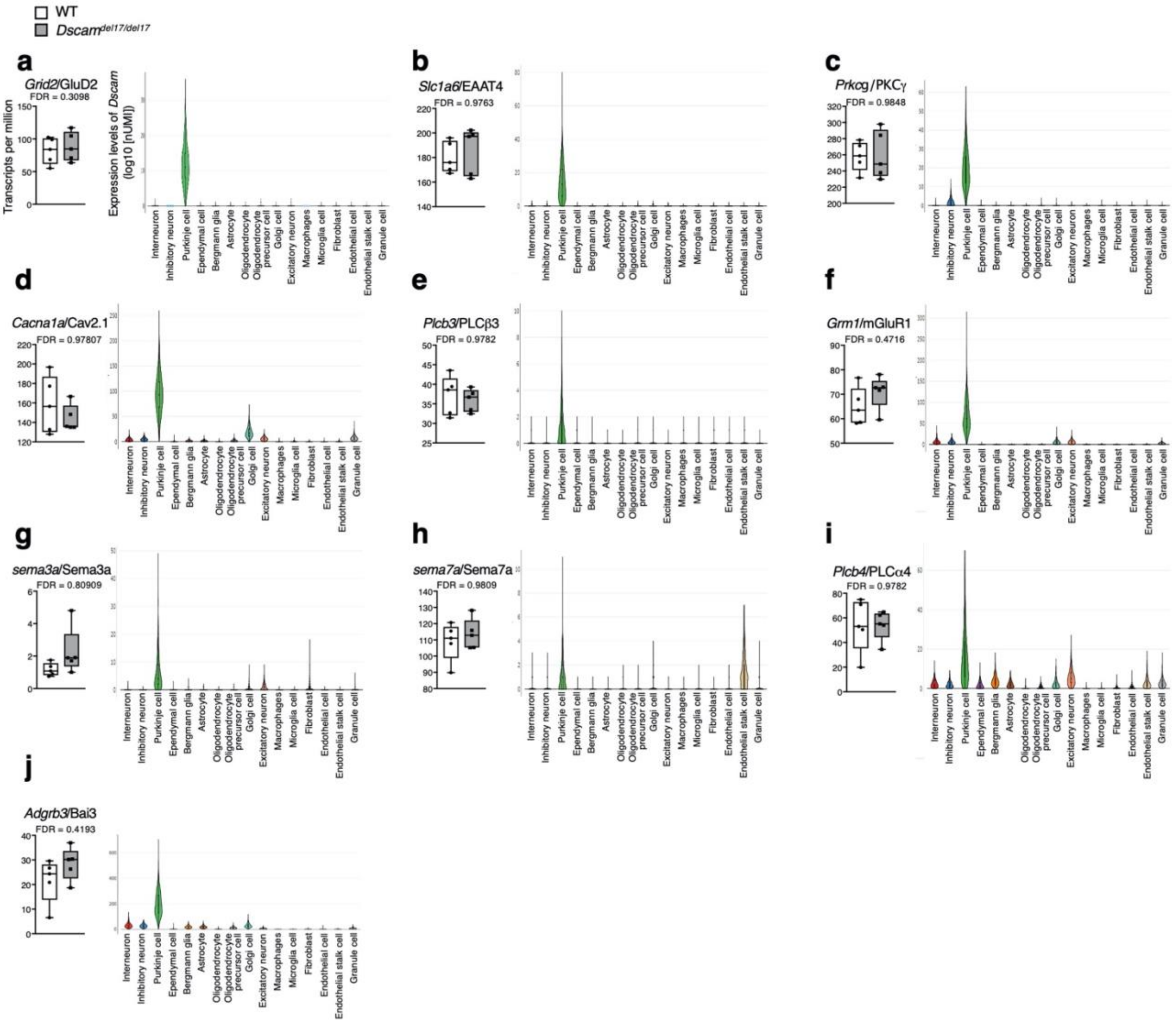

Supplementary Figure 8. Analyses of the expression of CF translocation-related genes via whole-cerebellum RNA-seq analyses and Purkinje cell-selective prominent expression.

a–j, Whole-cerebellum RNA-seq analyses using wild-type (WT) and *Dscam*$^{del17/del17}$ mice. *Dscam* mRNA was mainly expressed in Purkinje cells (Fig. 1a). Thus, the loss of DSCAM-mediated transcriptional activity[4] in *Dscam*$^{del17/del17}$ mice may significantly affect gene expression in Purkinje cells. The genes related to CF synaptogenesis, translocation, and elimination[5] were selected, and their expression was examined via whole-cerebellum RNA-seq analyses using wild-type (WT) and *Dscam*$^{del17/del17}$ mice (left in **a**–**i**): *Grid2* (gene name)/GluD2 (protein name) (**a**), *Slc1a6*/EAAT4 (**b**), *Prkcg*/PKCγ (**b**), *Cacna1a*/Cav2.1 (**d**), *Plcb3*/PLCβ3 (**e**), *Grm1*/mGluR1 (**f**), *sema3a*/Sema3a (**g**), *sema7a*/Sema7a (**h**), *Plcb4*/PLCα4 (**i**), and *Adgrb3*/Bai3 (**j**). Box plots show the median (horizontal line), quartiles (box), and range (whiskers); data were analysed via DEseq2 (*n* = 5 each). Purkinje cell-selective expression was confirmed by using the public single-cell portal site and dataset submitted by Kozareva et al[6] (right in **a**–**i**) as well as in Fig. 1a. Violin plot of log10(nUMI) per profile across the 16 cell types identified. WT, wild-type; FDR, false-discovery rate; UMI, unique molecular identifier.

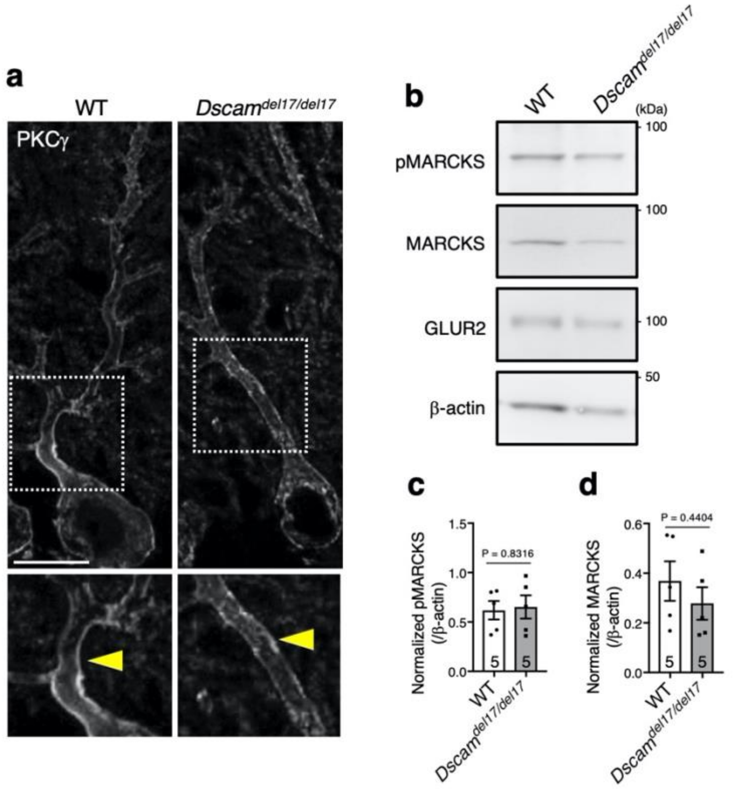

Supplementary Figure 9. No prominent change in the downstream molecules of mGluR1 in the *Dscam$^{del17/del17}$* cerebellum.

The activation of mGluR produces IP_3, which causes subsequent Ca^{2+} release through IP_3Rs and activation of Ca^{2+}-dependent PKC isoform (α, β, and γ). This signal transduction is essential for the elicitation of long-term depression (LTD) in the cerebellum. mGluR stimulation increased the phosphorylation of protein kinase Cγ (PKCγ) and a substrate of PKC, myristoylated alanine-rich C-kinase substrate (MARCKS), in Purkinje cells[7].

a, Immunohistochemical analyses using PKCγ antibody and the sagittal section of P30 wild-type (WT) and *Dscam$^{del17/del17}$* cerebella revealed that translocation of PKCγ to the Purkinje cell membrane was normal in *Dscam$^{del17/del17}$* mice. Higher magnification at the bottom represents the area surrounded by the white dotted box in the upper images. Yellow arrowheads indicate the subcellular localisation of PKCγ along the plasma membrane in Purkinje cell dendrites. Data are confirmed by three independent experiments. Scale bar, 20 μm.

b, Quantitative western blot analyses using MARCKS and phosphorylated MARCKS (Ser152/156) antibody and the lysates of P30 WT and *Dscam$^{del17/del17}$* cerebellum revealed that there was no significant quantitative difference between WT and *Dscam$^{del17/del17}$* mice. Data are confirmed by three independent experiments.

c–d, Quantitative western blot analyses using MARCKS and phosphorylated MARCKS (Ser152/156) antibody and the lysates of P30 WT and *Dscam$^{del17/del17}$* cerebellum revealed that there was no significant quantitative difference between WT and *Dscam$^{del17/del17}$* mice. The numbers in each column indicate the number of mice examined. Data represent mean ± SEM; unpaired two-tailed *t*-test.

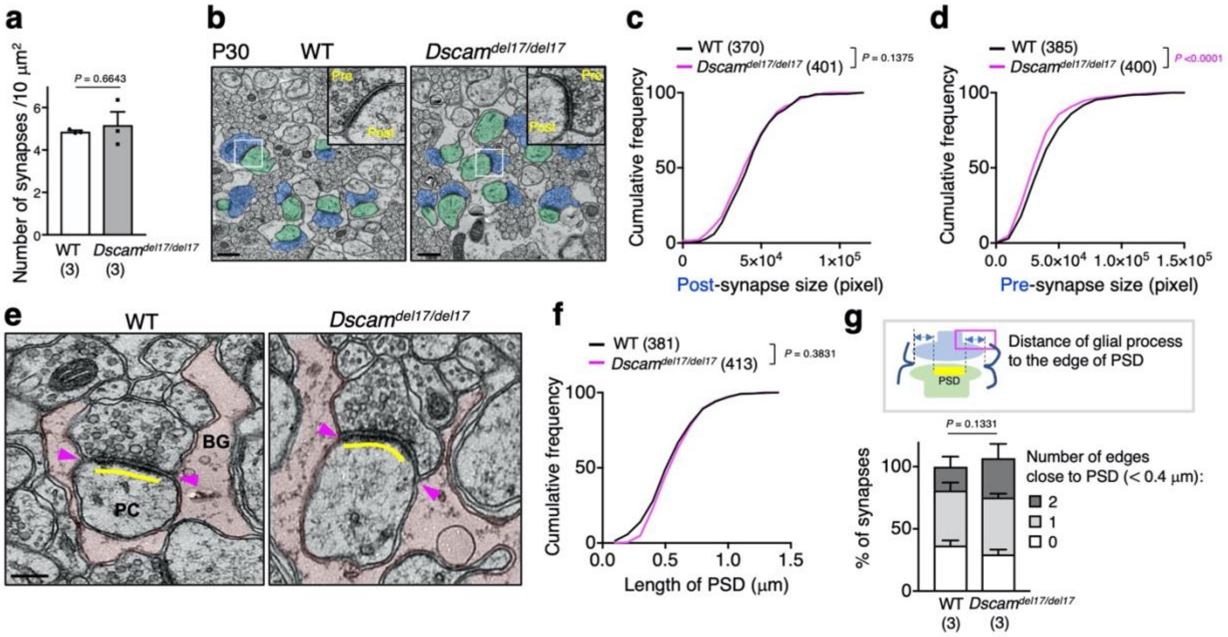

Supplementary Figure 10. Morphological analysis of the PF synapses in the *Dscam$^{del17/del17}$* cerebellum.

a, Quantitative analyses of synapses using electron microscopy for the number of synapses in the wild-type (WT) and *Dscam$^{del17/del17}$* mouse molecular layers. There was no significant quantitative difference between WT and *Dscam$^{del17/del17}$* mice. The numbers in parentheses are mice count. Data represent mean ± SEM; unpaired two-tailed *t*-test.

b, Transmission electron microscopy of WT and *Dscam$^{del17/del17}$* mouse molecular layers. Pre-synapses (Pre) and post-synapses (Post) are tinted blue and green, respectively. Higher magnification at the top represents the area surrounded by the white box in the larger image. Data are confirmed by three independent experiments. Scale bars, 500 nm.

c–d, Cumulative histogram of the size of post-synapse (**c**) and pre-synapse (**d**) from three littermates for each genotype. The numbers in parentheses are the number of synapses analysed. Two-tailed Mann-Whitney U test.

e, Transmission electron microscopy of WT and *Dscam$^{del17/del17}$* mouse molecular layers. The Bergmann glial (BG) processes are tinted pink. The postsynaptic density (PSD) is indicated by yellow lines. The closest BG processes are indicated by the pink arrowheads. Data are confirmed by three independent experiments. PC, Purkinje cell. Scale bar, 200 nm.

f, Cumulative histogram of PSD length. Samples were collected from three littermates of each genotype. There was no significant quantitative difference between WT and *Dscam$^{del17/del17}$* mice. The numbers in parentheses are the number of synapses analysed. Two-tailed Mann-Whitney U test.

g, Percentage of synapses classified by the distance between the edge of the PSD and BG processes. There was no significant quantitative difference between WT and *Dscam$^{del17/del17}$* mice. The numbers in parentheses are mice count. Data represent mean ± SEM; two-way ANOVA plus Tukey's multiple comparison test.

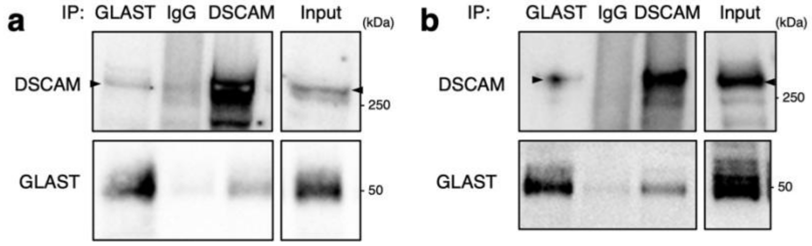

Supplementary Figure 11. Co-immunoprecipitation and western blot assays using the synaptosomal fraction.

a and **b**, Two independent analyses of immunoprecipitation analyses. The arrowheads indicate the bands of DSCAM. Input, 1% of total lysate in **a**, and 3% in **b**.

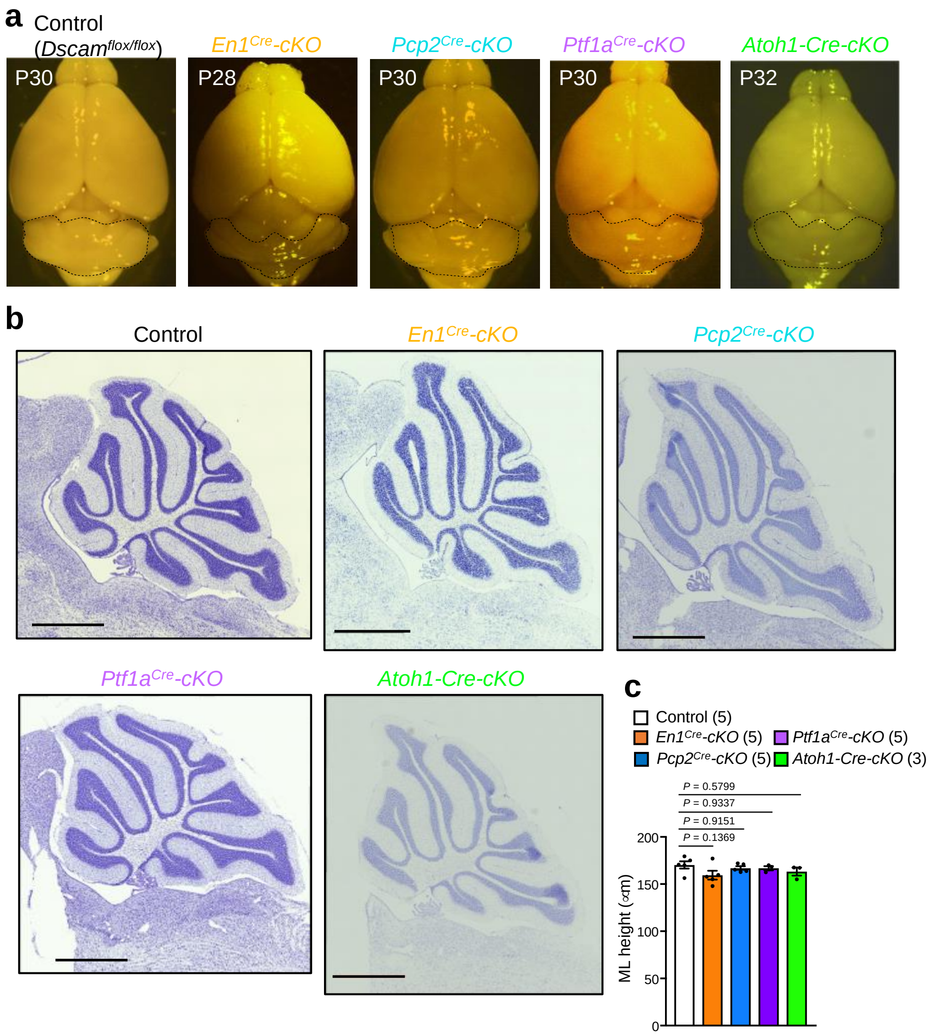

Supplementary Figure 12. Overall brain structure of *Dscam* cKO mice.

a, P28–30 control and *Dscam* cKO mouse brains. Since *En1*^{Cre}*-cKO* causes the depletion of *Dscam* transcripts in the caudo-dorsal midbrain and cerebellum[8, 9], prominent hypertrophy of the dorsal midbrain was observed in *En1*^{Cre}*-cKO*, as well as in *Dscam*$^{del17/del17}$ brains (Supplementary Figure 2d). The cerebellum (dotted line) of *En1*^{Cre}*-cKO* mice was pushed by the hypertrophic dorsal midbrain. Nevertheless, the whole cerebellar structure in all four cKO mice was normal. cKO, conditional knockout.

b, Nissl-stained sagittal sections of control and *Dscam* cKO cerebella did not reveal any gross anatomical differences. Data are confirmed by three independent experiments. Scale bars, 1.0 mm.

c, The height of the molecular layer in the sulcus of lobule 6 in all four cKO mice was normal. The numbers in parentheses are mice count. Data represent mean ± SEM; two-way ANOVA with multiple comparison.

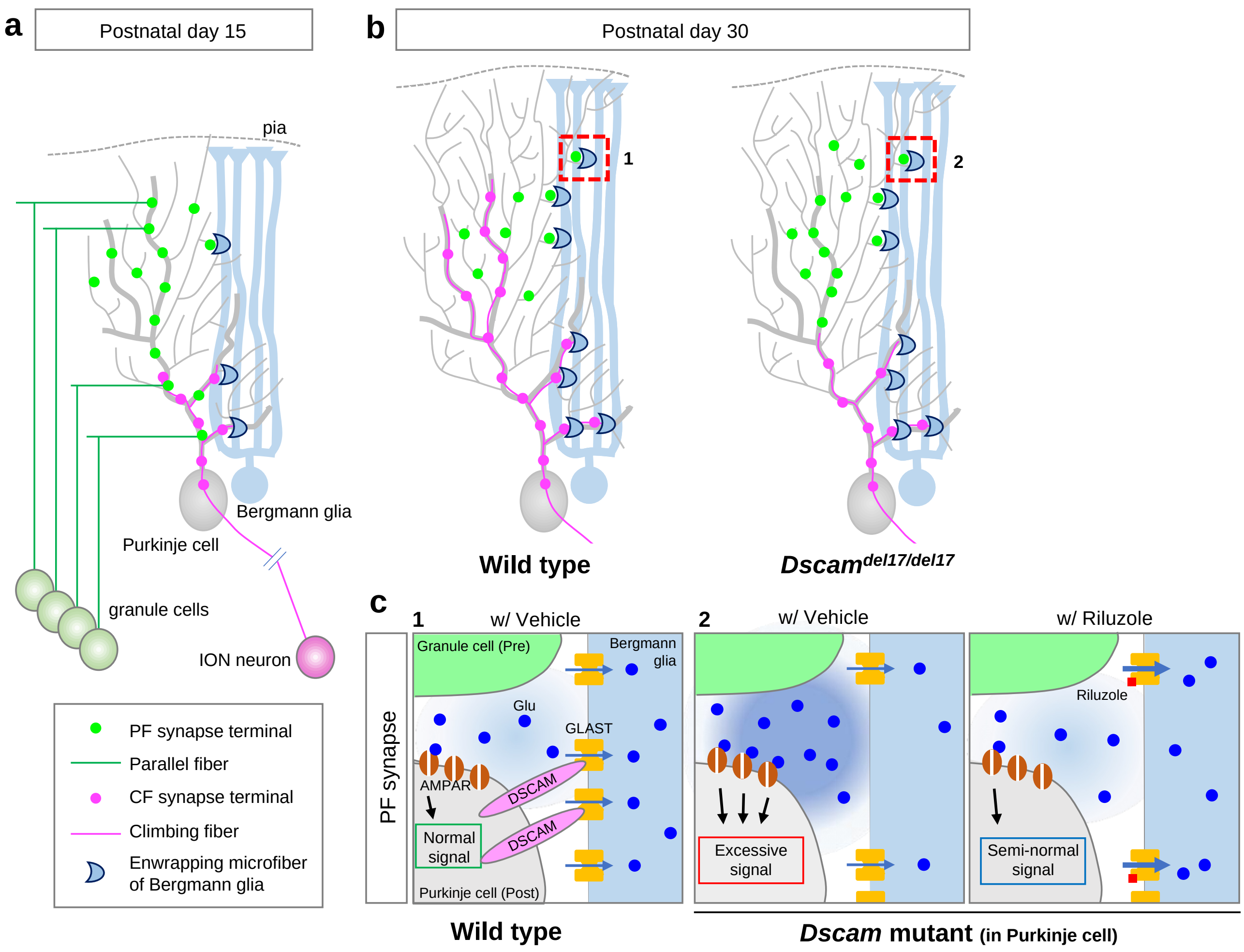

Supplementary Figure 13. Schematic diagram of the phenotype of *Dscam*-deficient mice and proposed molecular mechanisms.

a–b, Schematics depicting the positional relationship of CF and PF synapses on the dendrites of Purkinje cells around postnatal day 15 (P15) and 30 (P30). **a**, Inferior olive nucleus (ION) neuron and granule cells extend axons, named climbing fibres (CFs) and parallel fibres (PFs), on the Purkinje cell dendrite at molecular layers of the cerebellum. Until P15, CFs and PFs form synapses simultaneously on the proximal domain of the Purkinje cell dendrite. Bergmann glia enwrap these synapses from the proximal to the distal areas of the molecular layer. **b**, At P30, the territories of CF and PF innervation are spatially segregated, such that translocation of CFs makes more synapses on the middle domain of the Purkinje cell dendrite and PF synapses are eliminated in the wild-type cerebellum. In contrast, in *Dscam*$^{del17/del17}$ mice, translocation of CF synapses is impaired, and PF synapses are still observed in the proximal domain of Purkinje cell dendrites. Higher magnification in **c** (1 and 2) represents the area surrounded by the red dotted boxes.

c, Schematics depicting the function of the DSCAM-GLAST interaction between the Purkinje cell dendritic spine and enwrapping Bergmann glia in PF synapses in wild-type and *Dscam*$^{del17/del17}$ mice. GLAST in Bergmann glia interacts with DSCAM in the postsynaptic terminal (Post) of Purkinje cells intercellularly and localises and functions in the peri-synaptic area of PF synapses. GLAST transports glutamate (Glu)

from the synaptic cleft to the cytoplasm of Bergmann glia upon neural activation in the wild-type cerebellum. In contrast, in *Dscam*-deficient (especially in Purkinje cells) mice, the amount of GLAST in the peri-synaptic area is reduced. Increased residual glutamate in the synaptic cleft binds to glutamate receptors, such as AMPA receptor (AMPAR). This may cause unusual, excessive signals in the soma of Purkinje cells. Riluzole treatment activates glutamate transport of the residual GLAST so that the excessive signals in *Dscam*-deficient Purkinje cells are rescued. Pre, presynaptic terminal; Post, postsynaptic terminal; Glu, glutamate

Supplementary Table 1. Statistical analyses of major components in the $Dscam^{del17/del17}$ cerebellum.

Cleaved Caspase3 (whole lobule)	P7		P15		P30	
	WT	$Dscam^{del17/del17}$	WT	$Dscam^{del17/del17}$	WT	$Dscam^{del17/del17}$
Cell density (cm^2)	8.98 ± 3.49	10.41 ± 6.28	4.80 ± 0.68	3.54 ± 1.53	0 ± 0	0 ± 0
P value (Student's t-test)	0.853		0.488		N/A	
KI67 (P7, P15: IGL, P30: GCL)	P7		P15		P30	
	WT	$Dscam^{del17/del17}$	WT	$Dscam^{del17/del17}$	WT	$Dscam^{del17/del17}$
Cell density (100 μm^2)	5.65 ± 0.61	3.61 ± 2.27	0.14 ± 0.08	0 ± 0	0 ± 0	0 ± 0
P value (Student's t-test)	0.417		0.213		N/A	
KI67 (P7, P15: EGL, P30: GCL)	P7		P15		P30	
	WT	$Dscam^{del17/del17}$	WT	$Dscam^{del17/del17}$	WT	$Dscam^{del17/del17}$
Cell density (100 μm^2)	94.53 ± 0.56	109.99 ± 19.80	70.85 ± 4.69	95.85 ± 20.57	0 ± 0	0 ± 0
P value (Student's t-test)	0.518		0.280		N/A	
PAX6 (whole lobule)	P7		P15		P30	
	WT	$Dscam^{del17/del17}$	WT	$Dscam^{del17/del17}$	WT	$Dscam^{del17/del17}$
Cell density (100 μm^2)	56.12 ± 2.54	66.73 ± 5.73	48.06 ± 4.40	49.08 ± 1.65	51.29 ± 2.87	50.22 ± 5.03
P value (Student's t-test)	0.153		0.859		0.880	
OLIG2 (whole lobule)	P7		P15		P30	
	WT	$Dscam^{del17/del17}$	WT	$Dscam^{del17/del17}$	WT	$Dscam^{del17/del17}$
Cell density (100 μm^2)	179.40 ± 36.39	73.62 ± 2.96	92.82 ± 12.84	77.95 ± 9.64	49.69 ± 9.05	60.91 ± 15.44
P value (Student's t-test)	0.044		0.431		0.616	
IBAI (whole lobule)	P7		P15		P30	
	WT	$Dscam^{del17/del17}$	WT	$Dscam^{del17/del17}$	WT	$Dscam^{del17/del17}$
Cell density (μm^2)	175.07 ± 18.59	188.12 ± 2.96	196.00 ± 7.97	209.61 ± 33.26	157.40 ± 32.84	160.10 ± 15.44
P value (Student's t-test)	0.746		0.748		0.961	
Parvalbmin (ML)	P7		P15		P30	
	WT	$Dscam^{del17/del17}$	WT	$Dscam^{del17/del17}$	WT	$Dscam^{del17/del17}$
Cell density (100 μm^2)	1.81 ± 0.56	2.38 ± 0.15	4.83 ± 0.65	2.83 ± 0.06	3.71 ± 0.63	6.84 ± 1.69
P value (Student's t-test)	0.436		0.057		0.195	
PAX2 (P7, P15: whole lobule, P30: GCL)	P7		P15		P30	
	WT	$Dscam^{del17/del17}$	WT	$Dscam^{del17/del17}$	WT	$Dscam^{del17/del17}$
Cell density (100 μm^2)	5.02 ± 1.16	3.40 ± 1.00	5.09 ± 0.34	4.36 ± 1.29	3.67 ± 0.45	4.80 ± 0.51
P value (Student's t-test)	0.378		0.682		0.427	
vGAT (ML)	P7		P15		P30	
	WT	$Dscam^{del17/del17}$	WT	$Dscam^{del17/del17}$	WT	$Dscam^{del17/del17}$
Relative intensity (normarized by Calbindin)	2.56 ± 0.57	1.43 ± 0.38	0.66 ± 0.01	0.85 ± 0.06	1.64 ± 0.42	1.60 ± 0.48
P value (Student's t-test)	0.202		0.251		0.957	

Supplementary Table 2. A table summarising whether the experiment was performed in a blind (B), automatic measurement (Auto), or non-blind manner (NB).

B: blind, NB: not blind, Auto: Automatic Measurement

Figure 1	a	-	(Public data)
Figure 1	b	-	(Public data)
Figure 1	c	-	(WB images)
Figure 1	d	-	(Drawing)
Figure 1	e	-	(Microphotograph)
Figure 1	f	-	(WB images)
Figure 1	g	-	(Microphotograph)
Figure 1	h	-	(Microphotograph)
Figure 2	a	-	(Microphotograph)
Figure 2	b	-	(Microphotograph)
Figure 2	c	B	
Figure 2	d	Auto	
Figure 2	e	Auto	
Figure 2	f	Auto	
Figure 3	a	-	(Current trace)
Figure 3	b	NB	
Figure 3	c	NB	
Figure 3	d	NB	
Figure 3	e	-	(Current trace)
Figure 3	f	NB	
Figure 3	g	NB	
Figure 3	h	-	(Current trace)
Figure 3	i	NB	
Figure 3	j	NB	
Figure 4	k	-	(Microphotograph)
Figure 5	l	B	
Figure 6	m	B	
Figure 4	a	-	(WB images)
Figure 4	b	-	(WB images)
Figure 4	c	-	(Drawing)
Figure 4	d	-	(WB images)
Figure 4	e	-	(Microphotograph)
Figure 5	a	-	(Table)
Figure 5	b	-	(Microphotograph)
Figure 5	c	Auto	
Figure 5	d	Auto	
Figure 5	e	Auto	
Figure 6	a	-	(Drawing)
Figure 6	b	-	(Microphotograph)
Figure 6	c	B	
Figure 7	a	-	(Drawing)
Figure 7	b	-	(Current trace)
Figure 7	c	Auto	
Figure 7	d	Auto	

Supplementary Figure 1	a	-	(Public data)
Supplementary Figure 1	b	Auto	
Supplementary Figure 1	c	-	(Microphotograph)
Supplementary Figure 1	d	-	(Microphotograph)
Supplementary Figure 2	a	-	(Drawing)
Supplementary Figure 2	b	-	(Drawing)
Supplementary Figure 2	c	-	(WB images)
Supplementary Figure 2	d	-	(Digital photograph)
Supplementary Figure 2	e	-	(Microphotograph)
Supplementary Figure 2	f	-	(Microphotograph)
Supplementary Figure 2	g	-	(Microphotograph)
Supplementary Figure 3	a	-	(Digital photograph)
Supplementary Figure 3	b	-	(Microphotograph)
Supplementary Figure 3	c	Auto	
Supplementary Figure 3	d	Auto	
Supplementary Figure 3	e	Auto	
Supplementary Figure 4	a	-	(Microphotograph)
Supplementary Figure 4	b	Auto	
Supplementary Figure 4	c	-	(Microphotograph)
Supplementary Figure 4	d	Auto	
Supplementary Figure 4	e	-	(Microphotograph)
Supplementary Figure 5	a-h	-	(Microphotograph)
Supplementary Figure 6	a-l	NB	
Supplementary Figure 7	a	Auto	
Supplementary Figure 7	b	-	(WB images)
Supplementary Figure 7	c	Auto	
Supplementary Figure 7	d	-	(Microphotograph)
Supplementary Figure 8	a-h	Auto	
Supplementary Figure 9	a	-	(Microphotograph)
Supplementary Figure 9	b	-	(WB images)
Supplementary Figure 9	c	Auto	
Supplementary Figure 9	d	Auto	

Supplementary Figure 10	a	B	
Supplementary Figure 10	b	-	(Microphotograph)
Supplementary Figure 10	c	B	
Supplementary Figure 10	d	B	
Supplementary Figure 10	e	-	(Microphotograph)
Supplementary Figure 10	f	B	
Supplementary Figure 10	g	NB	
Supplementary Figure 11	a	-	(WB images)
Supplementary Figure 11	b	-	(WB images)
Supplementary Figure 12	a	-	(Digital photograph)
Supplementary Figure 12	b	-	(Microphotograph)
Supplementary Figure 12	c	Auto	
Supplementary Figure 13	a	-	(Drawing)
Supplementary Figure 13	b	-	(Drawing)
Supplementary Figure 13	c	-	(Drawing)
Supplementary Table 1		NB	

Supplementary References

1. Wizeman, J. W., Guo, Q., Wilion, E. M. & Li, J. Y. Specification of diverse cell types during early neurogenesis of the mouse cerebellum. *eLife* **8**, e42388 (2019). 10.7554/eLife.42388, Pubmed:30735127.

2. Arimura, N. et al. DSCAM regulates delamination of neurons in the developing midbrain. *Sci. Adv.* **6**, eaba1693 (2020). 10.1126/sciadv.aba1693, Pubmed:32917586.

3. Fuerst, P. G., Koizumi, A., Masland, R. H. & Burgess, R. W. Neurite arborization and mosaic spacing in the mouse retina require DSCAM. *Nature* **451**, 470-474 (2008). 10.1038/nature06514, Pubmed:18216855.

4. Sachse, S. M. et al. Nuclear import of the DSCAM-cytoplasmic domain drives signaling capable of inhibiting synapse formation. *EMBO J.* **38**, e99669 (2019). 10.15252/embj.201899669, Pubmed:30745319.

5. Kano, M. & Watanabe, T. Developmental synapse remodeling in the cerebellum and visual thalamus. *F1000Res*. **8** (2019). 10.12688/f1000research.18903.1, Pubmed:31372212.

6. Kozareva, V. et al. A transcriptomic atlas of mouse cerebellar cortex comprehensively defines cell types. *Nature* **598**, 214-219 (2021). 10.1038/s41586-021-03220-z, Pubmed:34616064.

7. Sugawara, T., Hisatsune, C., Miyamoto, H., Ogawa, N. & Mikoshiba, K. Regulation of spinogenesis in mature Purkinje cells via mGluR/PKC-mediated phosphorylation of CaMKIIβ. *Proc. Natl Acad. Sci. U. S. A.* **114**, E5256-E5265 (2017). 10.1073/pnas.1617270114, Pubmed:28607044.

8. Sgaier, S. K. et al. Genetic subdivision of the tectum and cerebellum into functionally related regions based on differential sensitivity to engrailed proteins. *Development* **134**, 2325-2335 (2007). 10.1242/dev.000620, Pubmed:17537797.

9. Hashimoto, R. et al. Origins of oligodendrocytes in the cerebellum, whose development is controlled by the transcription factor, Sox9. *Mech. Dev.* **140**, 25-40 (2016). 10.1016/j.mod.2016.02.004, Pubmed:26940020.
