# Supplementary material for: Neuronal DSCAM regulates the peri-synaptic localization of GLAST in Bergmann glia for functional synapse formation
Source: Nat Commun. 2024 Feb 1;15:458. doi: 10.1038/s41467-023-44579-z (PMC10834496; doi:10.1038/s41467-023-44579-z)
Supplement: Supplementary file 3 — Reporting Summary [file 41467_2023_44579_MOESM3_ESM.pdf]

nature portfolio

Corresponding author(s): Nariko Arimura, Mikio Hoshino

Last updated by author(s): Nov 30, 2023

Reporting Summary

Nature Portfolio wishes to improve the reproducibility of the work that we publish. This form provides structure for consistency and transparency in reporting. For further information on Nature Portfolio policies, see our Editorial Policies and the Editorial Policy Checklist.

Statistics

For all statistical analyses, confirm that the following items are present in the figure legend, table legend, main text, or Methods section.

n/a	Confirmed	
☐	☒	The exact sample size (*n*) for each experimental group/condition, given as a discrete number and unit of measurement
☐	☒	A statement on whether measurements were taken from distinct samples or whether the same sample was measured repeatedly
☐	☒	The statistical test(s) used AND whether they are one- or two-sided<br>*Only common tests should be described solely by name; describe more complex techniques in the Methods section.*
☒	☐	A description of all covariates tested
☐	☒	A description of any assumptions or corrections, such as tests of normality and adjustment for multiple comparisons
☐	☒	A full description of the statistical parameters including central tendency (e.g. means) or other basic estimates (e.g. regression coefficient) AND variation (e.g. standard deviation) or associated estimates of uncertainty (e.g. confidence intervals)
☒	☐	For null hypothesis testing, the test statistic (e.g. *F*, *t*, *r*) with confidence intervals, effect sizes, degrees of freedom and *P* value noted<br>*Give P values as exact values whenever suitable.*
☒	☐	For Bayesian analysis, information on the choice of priors and Markov chain Monte Carlo settings
☒	☐	For hierarchical and complex designs, identification of the appropriate level for tests and full reporting of outcomes
☒	☐	Estimates of effect sizes (e.g. Cohen's *d*, Pearson's *r*), indicating how they were calculated

Our web collection on statistics for biologists contains articles on many of the points above.

Software and code

Policy information about availability of computer code

Data collection	Fluorescence imaging was performed using a Zeiss LSM 780 confocal microscope system (Carl Zeiss, Oberkochen, Germany) and ZEN 2009 software (Carl Zeiss) or spinning disk confocal super-resolution microscope (SpinSR10, Olympus Corporation, Tokyo, Japan) and cellSens Dimension (Ver 3.1.1, Olympus Corporation). To detect DSCAM-ALFA at both low and high magnifications, a Leica Stellaris 5 laser scanning confocal microscope (Leica, Wetzlar, Germany) was used and images were obtained with the LASX software (Leica). For high magnification, the Lightning 3D deconvolution method was used for image acquisition. Nissle staining data were corrected by all in one fluorescent microscopy BZ700 (Keyence). Quantification of the fluorescence intensity of immunolabeled cells was performed using the "Measure" and "Plot Profile" functions of ImageJ v1.46r. The number of cells, dendritic height, and the height and number of vGluT2 puncta were measured using Fiji/ImageJ 2.3.0/1.53. Electron microscopy data corrected by TEM, JEM-3200FS. WB data collected by ImageQuant LAS-4000 mini (Fujifilm). RNA-seq libraries were sequenced using Illumina NovaSeq platforms. Adapter and low-quality sequences were trimmed using Trimmomatic (version 0.36), and the trimmed reads were aligned to the reference mouse genome (GRCm38/mm10) using HISAT2 (version 2.2.0). Genome-wide expression levels were measured as a unit of transcripts per kilobase million (TPM) using StringTie (version 1.3.6) and the number of reads was counted per gene per sample using htseq-count within HTSeq (version 0.9.1). Differentially expressed genes were identified using DESeq2 (version 1.8.2). For gene ontology analysis, DAVID Bioinformatics Resources (version 6.8) was used (National Institute of Allergy and Infectious Diseases, National Institutes of Health; https://david.ncifcrf.gov). Current responses in electrophysiological analyses were recorded using an Axopatch 200 B amplifier, and the pCLAMP system (v9.2) was used for data acquisition and analysis. Statistical analyses were performed using Prism v7.0 (GraphPad Software, La Jolla, CA, USA) or R Studio v3.0 (R-Tools Technology, Boston, MA, USA).
Data analysis	All statistical analyses were preformed by ImageJ (Open source), Microsoft Excel (Microsoft), and Prism V8 (GraphPad).

For manuscripts utilizing custom algorithms or software that are central to the research but not yet described in published literature, software must be made available to editors and reviewers. We strongly encourage code deposition in a community repository (e.g. GitHub). See the Nature Portfolio guidelines for submitting code & software for further information.

Data

Policy information about availability of data

All manuscripts must include a data availability statement. This statement should provide the following information, where applicable:

- Accession codes, unique identifiers, or web links for publicly available datasets
- A description of any restrictions on data availability
- For clinical datasets or third party data, please ensure that the statement adheres to our policy

The following accession number is currently open: GSE190010. The data that support the findings of this study are available from the corresponding author upon reasonable request.

Research involving human participants, their data, or biological material

Policy information about studies with human participants or human data. See also policy information about sex, gender (identity/presentation), and sexual orientation and race, ethnicity and racism.

Reporting on sex and gender	Not applicable to this study
Reporting on race, ethnicity, or other socially relevant groupings	Not applicable to this study
Population characteristics	Not applicable to this study
Recruitment	Not applicable to this study
Ethics oversight	Not applicable to this study

Note that full information on the approval of the study protocol must also be provided in the manuscript.

Field-specific reporting

Please select the one below that is the best fit for your research. If you are not sure, read the appropriate sections before making your selection.

[x] Life sciences [ ] Behavioural & social sciences [ ] Ecological, evolutionary & environmental sciences

For a reference copy of the document with all sections, see nature.com/documents/nr-reporting-summary-flat.pdf

Life sciences study design

All studies must disclose on these points even when the disclosure is negative.

Sample size	Sample size was determined to be adequate based on similar sample sizes that have previously reported using similar experiments reported in references of this manuscript.<br>Ichikawa, R. et al. Territories of heterologous inputs onto Purkinje cell dendrites are segregated by mGluR1-dependent parallel fibre synapse elimination. Proc. Natl Acad. Sci. U. S. A. 113, 2282-2287 (2016). 10.1073/pnas.1511513113, Pubmed:26858447.<br>Watase, K. et al. Motor discoordination and increased susceptibility to cerebellar injury in GLAST mutant mice. Eur. J. Neurosci. 10, 976-988 (1998). 10.1046/j.1460-9568.1998.00108.x, Pubmed:9753165.<br>Watase, K. et al. Motor discoordination and increased susceptibility to cerebellar injury in GLAST mutant mice. Eur. J. Neurosci. 10, 976-988 (1998). 10.1046/j.1460-9568.1998.00108.x, Pubmed:9753165.<br>Chaudhry, F. A. et al. Glutamate transporters in glial plasma membranes: highly differentiated localizations revealed by quantitative ultrastructural immunocytochemistry. Neuron 15, 711-720 (1995). 10.1016/0896-6273(95)90158-2, Pubmed:7546749.<br>Yamagata, M. & Sanes, J. R. Dscam and Sidekick proteins direct lamina-specific synaptic connections in vertebrate retina. Nature 451, 465-469 (2008). 10.1038/nature06469, Pubmed:18216854.<br>Arimura, N. et al. DSCAM regulates delamination of neurons in the developing midbrain. Sci. Adv. 6 (2020). 10.1126/sciadv.aba1693, Pubmed:32917586.<br>Fuerst, P. G., Koizumi, A., Masland, R. H. & Burgess, R. W. Neurite arborization and mosaic spacing in the mouse retina require DSCAM. Nature 451, 470-474 (2008). 10.1038/nature06514, Pubmed:18216855.<br>Ageta-Ishihara, N. et al. A CDC42EP4/septin-based perisynaptic glial scaffold facilitates glutamate clearance. Nat. Commun. 6, 10090 (2015). 10.1038/ncomms10090, Pubmed:26657011.<br>Takayasu, Y., Iino, M., Shimamoto, K., Tanaka, K. & Ozawa, S. Glial glutamate transporters maintain one-to-one relationship at the climbing fiber-Purkinje cell synapse by preventing glutamate spillover. J. Neurosci. 26, 6563-6572 (2006). 10.1523/JNEUROSCI.5342-05.2006, Pubmed:16775144.
Data exclusions	No other data were excluded from the analyses.
Replication	For each experimental condition, at least 3 sections were analyzed per animal and at least 3 animals were used per conditions. All attempts at replication were successful expect for the failures due to sickness or death of animals during the experimental period.

Randomization	Mice and samples were randomized from each group.
Blinding	In most of our experiments, especially in the electro physiological and mouse behavioral experiments, it was difficult to perform them in a blinded manner, because a single researcher with advanced skills had to concentrate on a single experiment. For general immunostaining and western blotting, a single researcher was in charge throughout a single experiment but the data from the second and third experiments were analyzed by a different researcher to confirm the reproducibility of the results. Furthermore, in most cases, we measured the data automatically by using some detectors or microscopy. A table summarizing whether the experiment was performed in a blind, automatic measurement, or non-blind manner is provided as Supplementary Table 2 in the supplementary information.

Reporting for specific materials, systems and methods

We require information from authors about some types of materials, experimental systems and methods used in many studies. Here, indicate whether each material, system or method listed is relevant to your study. If you are not sure if a list item applies to your research, read the appropriate section before selecting a response.

Materials & experimental systems

n/a	Involved in the study	
☐	☒	Antibodies
☐	☒	Eukaryotic cell lines
☒	☐	Palaeontology and archaeology
☐	☒	Animals and other organisms
☒	☐	Clinical data
☒	☐	Dual use research of concern
☒	☐	Plants

Methods

n/a	Involved in the study	
☒	☐	ChIP-seq
☒	☐	Flow cytometry
☒	☐	MRI-based neuroimaging

Antibodies

Antibodies used

Antibody list
1. chick anti-GFP (1:500; ab13970; Abcam, Cambridge, UK)
2. goat anti-Calbindin (1:500; AB_2571569; Frontier Institute, Hokkaido, Japan)
3. guinea pig anti-vGluT2 (1:500; AB_2571621; Frontier Institute)
4. goat anti-vGluT2 (1:500; AB_2571620; Frontier Institute)
5. guinea pig anti-vGluT1 (1:500; AB5905; Millipore)
6. guinea pig anti-VGAT (1;500; AB_2571624; Frontier Institute)
7. rat anti-GFAP (1:500; 13-0300; Thermo Fisher Scientific, Waltham, MA, USA)
8. goat anti-GLAST (1:500; AB_2571716; Frontier Institute)
9. rabbit anti-PKCγ (1:500; AB_2571824; Frontier Institute)
10. rabbit anti-DSCAM (1:100–10000; LS-B5787-50; LifeSpan Biosystems, Minneapolis, MN, USA)
11. mouse anti-DSCAM (1:100–10000; MAB2603; Millipore)
12. rabbit anti-DSCAM (1:100–10000; NBP2-30716; Novus Biologicals, Centennial CO, USA)
13. rabbit anti-DSCAM (1:100–10000; ORB156648; Biorbyt, Cambridge, UK)
14. rabbit anti-DSCAM (1:100–10000; sc79437; Santa Cruz Biotechnology, Santa Cruz, CA, USA)
15. rabbit anti-DSCAM (1:100–10000; HPA019324; Sigma-Aldrich, St. Louis, MO, USA)
16. goat anti-DSCAM (1:100–10000; AF3666; R&D Systems, Minneapolis, MN, USA)
17. rabbit anti-DSCAM antibody produced in our laboratory (antigen: 1880-2008 amino acids)
18. goat anti-RORα (1:1,000, sc-6062; Santa Cruz Biotechnology)
19. rabbit anti-β-actin (1:1,000, sc-47778; Santa Cruz Biotechnology)
20. rabbit anti-EAAT1/GLAST (1:500; ab416; Abcam)
21. rabbit anti-GluD2 (1:500, AB_2571600; Frontier Institute)
22. mouse anti-PSD95 (1:1,000, MA1-045; Invitrogen)
23. rabbit anti-Cul3 (1:1,000, ab72187; Abcam)
24. rabbit anti-GFP (1:1,000, #598; MBL, Woburn, MA, USA)
25. rabbit anti-Flag (1:1,000, SAB4301135; Sigma-Aldrich)
26. rabbit anti-pMARCKS (Ser152/156) (1:1,000, #07-1238; Millipore)
27. mouse anti-MARCKS (1:1,000, WH0004082M6; Sigma-Aldrich)
28. anti-mouse IgG (1:1,000, PI-2000; Vector Laboratories, Burlingame, CA, USA)
29. anti-rabbit IgG (1:1,000, PI-1000; Vector Laboratories)
30. anti-goat IgG (1:1,000, PI-9500; Vector Laboratories)
31. rabbit IgG (011-000-003; Jackson ImmunoResearch Laboratories
32. rabbit anti-ALFA (N1581; NanoTag Biotechnologies)
33. rabbit anti-EAAT1/GLAST (#5684; Cell signaling technologies)
34. rabbit IgG (PM035; MBL, Tokyo, Japan)
35. AffiPure F(ab')2 Fragment conjugated with Donkey Alexa Fluor 488, 568, 594, or 647 (1:1,000; Jackson ImmunoResearch Laboratories, Baltimore Pile, PA, USA)

36. Alexa Fluor®   (1:1,000; Abcam)
37. FluoTag-X2 anti-ALFA conjugated Alexa647 (N1502-AF647-L; NanoTag Biotechnologies, Gottingen, Germany
38. Nanogold-IgG rabbit anti-goat IgG(H+L) (2005; Nanoprobes,Yaphank, NY, USA)

Validation

Validation
All commercial antibodies are available and validated by the manufacture as suitable for the applications. The product size in Western Blot experiments were comparing the target to protein size ladder.
1. chick anti-GFP (1:500; ab13970; Abcam, Cambridge, UK)
https://www.abcam.co.jp/products/primary-antibodies/gfp-antibody-ab13970.html
2. goat anti-Calbindin (1:500; AB_2571569; Frontier Institute, Hokkaido, Japan)
https://www.antibodyregistry.org/AB_2571569
3. guinea pig anti-vGluT2 (1:500; AB_2571621; Frontier Institute)
https://www.antibodyregistry.org/AB_2571621
4. goat anti-vGluT2 (1:500; AB_2571620; Frontier Institute)
https://www.antibodyregistry.org/AB_2571620
5. guinea pig anti-vGluT1 (1:500; AB5905; Millipore)
https://www.merckmillipore.com/JP/ja/product/Anti-Vesicular-Glutamate-Transporter-1-Antibody,MM_NF-AB5905
6. guinea pig anti-VGAT (1;500; AB_2571624; Frontier Institute)
https://www.antibodyregistry.org/AB_2571624
7. rat anti-GFAP (1:500; 13-0300; Thermo Fisher Scientific, Waltham, MA, USA)
https://www.thermofisher.com/antibody/product/GFAP-Antibody-clone-2-2B10-Monoclonal/13-0300
8. goat anti-GLAST (1:500; AB_2571716; Frontier Institute)
https://www.antibodyregistry.org/AB_2571716
9. rabbit anti-PKCγ (1:500; AB_2571824; Frontier Institute)
https://www.antibodyregistry.org/AB_2571824
10. rabbit anti-DSCAM (1:100–10000; LS-B5787-50; LifeSpan Biosystems, Minneapolis, MN, USA)
https://www.lsbio.com/antibodies/dscam-antibody-aa1548-1597-ihc-wb-western-ls-b5787/141192
11. mouse anti-DSCAM (1:100–10000; MAB2603; Millipore)
https://www.merckmillipore.com/INTERSHOP/web/WFS/Merck-INTL-Site/es_ES/-/USD/ShowDocument-File?ProductSKU=MM_NF-MAB2603&DocumentId=null&DocumentType=COA&Language=EN&Country=US&ProductBatchNo=NG1872071&Origin=PDP
12. rabbit anti-DSCAM (1:100–10000; NBP2-30716; Novus Biologicals, Centennial CO, USA)
https://www.novusbio.com/products/dscam-antibody_nbp2-30716
13. rabbit anti-DSCAM (1:100–10000; ORB156648; Biorbyt, Cambridge, UK)
https://www.biorbyt.com/dscam-antibody-orb156648.html
14. rabbit anti-DSCAM (1:100–10000; sc79437; Santa Cruz Biotechnology, Santa Cruz, CA, USA)
https://www.santa-cruz.ru/item-280311.html
15. rabbit anti-DSCAM (1:100–10000; HPA019324; Sigma-Aldrich, St. Louis, MO, USA)
https://www.sigmaaldrich.com/JP/ja/product/sigma/hpa019324
16. goat anti-DSCAM (1:100–10000; AF3666; R&D Systems, Minneapolis, MN, USA)
https://www.rndsystems.com/products/human-dscam-long-isoform-antibody_af3666
17. rabbit anti-DSCAM antibody produced in our laboratory (antigen: 1880-2008 amino acids)
This antibody was validated by western blot analyses, but not by immunohistochemical analyses using cerebella brain slices in our lab.
18. goat anti-RORα (1:1,000, sc-6062; Santa Cruz Biotechnology)
https://datasheets.scbt.com/sc-6062.pdf
19. rabbit anti-β-actin (1:1,000, sc-47778; Santa Cruz Biotechnology)
https://datasheets.scbt.com/sc-47778.pdf
20. rabbit anti-EAAT1/GLAST (1:500; ab416; Abcam)
https://www.abcam.co.jp/products/primary-antibodies/eaat1-antibody-ab416.html
21. rabbit anti-GluD2 (1:500, AB_2571600; Frontier Institute)
https://www.antibodyregistry.org/AB_2571600
22. mouse anti-PSD95 (1:1,000, MA1-045; Invitrogen)
https://www.thermofisher.com/antibody/product/PSD-95-Antibody-clone-6G6-1C9-Monoclonal/MA1-045
23. rabbit anti-Cul3 (1:1,000, ab72187; Abcam)
https://www.abcam.co.jp/products/primary-antibodies/cullin-3cul-3-antibody-ab72187.html
24. rabbit anti-GFP (1:1,000, #598; MBL, Woburn, MA, USA)
https://ruo.mbl.co.jp/bio/dtl/A/index.html?pcd=598
25. rabbit anti-Flag (1:1,000, SAB4301135; Sigma-Aldrich)
https://www.sigmaaldrich.com/JP/ja/product/sigma/sab4301135
26. rabbit anti-pMARCKS (Ser152/156) (1:1,000, #07-1238; Millipore)
https://www.merckmillipore.com/JP/ja/product/Anti-phospho-MARCKS-Ser152-156-Antibody,MM_NF-07-1238
27. mouse anti-MARCKS (1:1,000, WH0004082M6; Sigma-Aldrich)
https://www.sigmaaldrich.com/JP/ja/product/sigma/wh0004082m6
28. anti-mouse IgG (1:1,000, PI-2000; Vector Laboratories, Burlingame, CA, USA)
https://vectorlabs.com/products/peroxidase-horse-anti-mouse-igg/
29. anti-rabbit IgG (1:1,000, PI-1000; Vector Laboratories)
https://vectorlabs.com/products/peroxidase-goat-anti-rabbit-igg/
30. anti-goat IgG (1:1,000, PI-9500; Vector Laboratories)
https://vectorlabs.com/products/peroxidase-horse-anti-goat-igg/

31. rabbit IgG (011-000-003; Jackson ImmunoResearch Laboratories
https://www.jacksonimmuno.com/catalog/products/011-000-003
32. rabbit anti-ALFA (N1581; NanoTag Biotechnologies)
https://sysy.com/product/N1581
33. rabbit anti-EAAT1/GLAST (#5684; Cell signaling technologies)
https://www.cellsignal.jp/products/primary-antibodies/eaat1-d44e2-xp-rabbit-mab/5684
34. rabbit IgG (PM035; MBL, Tokyo, Japan)
https://ruo.mbl.co.jp/bio/dtl/A/index.html?pcd=PM035
35. AffiPure F(ab')2 Fragment conjugated with Donkey Alexa Fluor 488, 568, 594, or 647 (1:1,000; Jackson ImmunoResearch Laboratories, Baltimore Pile, PA, USA)
https://www.jacksonimmuno.com/catalog/products
36. Alexa Fluor® (1:1,000; Abcam)
https://www.abcam.co.jp/products?selected.classification=Secondary+antibodies--Alexa+Fluor%c2%ae+conjugated+secondaries
37. FluoTag-X2 anti-ALFA conjugated Alexa647 (N1502-AF647-L; NanoTag Biotechnologies, Gottingen, Germany
https://sysy.com/product/N1502-AF647-L
38. Nanogold-IgG rabbit anti-goat IgG(H+L) (2005; Nanoprobes,Yaphank, NY, USA)
https://www.universalbiologicals.com/nanogold-igg-rabbit-anti-goat-igg-h-l-min-x-hu-sr-prot-2005-grp

Eukaryotic cell lines

Policy information about cell lines and Sex and Gender in Research

Cell line source(s)	COS-7 cells (male) were obtained from RIKEN Cell Bank, RCB0539.
Authentication	We did not conduct further authentication for cells obtained from RIKEN. However, it is worth nothing that RIKEN authenticate their cell lines through STR analysis, as indicated on their product specification webpage.
Mycoplasma contamination	This cell line was confirmed to be negative for mycoplasma contamination by PCR.
Commonly misidentified lines (See ICLAC register)	No commonly misidentified cell lines were not used in the study.

Animals and other research organisms

Policy information about studies involving animals; ARRIVE guidelines recommended for reporting animal research, and Sex and Gender in Research

Laboratory animals	Species: mixed (C57BL/6 and B6C3F1); DscamALFA/ALFA, and C57BL/6; Pcp2Cre-cKO, Ptf1aCre-cKO, Tg-Atoh1-Cre-cKO; Sex: n/a; Age:between postnatal day 0 and 90.<br>Transgenic lines in mixed (BALB/cCrSlc and C57BL/6) backgrounds: Dscamdel17/ del17, En1Cre-cKO; Sex: n/a; Age: between postnatal day 0 and 90.<br>All mice were housed on a 12-h light/dark cycle with controlled room temperature (23±2°C), humidity (40-60%), free access to food and water.
Wild animals	The study did not involve wild animals.
Reporting on sex	In this study, the birth rate of mice (especially Dscamdel17/ del17 and En1Cre-cKO) was low, making it difficult to secure a cohesive number of animals of either sex. Therefore, male and female animals were used in the experiment without distinguishing between the two sexes.
Field-collected samples	The study did not involve samples collected from the field.
Ethics oversight	All animal experiments in this study were approved by the Animal Care and Use Committee of the National Institute of Neuroscience, NCNP, Japan (#2019027).

Note that full information on the approval of the study protocol must also be provided in the manuscript.

Plants

Seed stocks	Not applicable to this study
Novel plant genotypes	Not applicable to this study
Authentication	Not applicable to this study
